# Incorporating peer feedback in academic writing: a systematic review of benefits and challenges

**DOI:** 10.3389/fpsyg.2024.1506725

**Published:** 2024-11-26

**Authors:** Yuzhu Wei, Donghong Liu

**Affiliations:** School of Foreign Languages, Southeast University, Nanjing, China

**Keywords:** academic writing, peer feedback, affective benefits, benefits and challenges, psychological mindset

## Abstract

Academic writing is paramount to students’ academic success in higher education. Given the widely acknowledged benefits of peer feedback in diverse learning contexts, such as fostering a positive psychological mindset, there has been a growing interest in applying this approach to facilitate the development of academic writing. This study is launched to examine the primary features and findings of the studies that have investigated the benefits and challenges of the utilization of peer feedback in academic writing development. The methodology of this study incorporates a rigorous literature search methodology, encompassing database search, reference search, and manual search, which is subsequently followed by a content analysis of the selected studies. With the guidance of PRISMA 2020, a total of 60 related articles, spanning the period from 2014 to 2024, are selected through title screening, abstract screening and content screening, adhering to strict inclusion and exclusion criteria. The findings of this study reveal a growing global interest in peer feedback in academic writing, and highlight the need for future research on masters’/doctoral students and quantitative approaches to deepen understanding of its effects. Moreover, 16 distinct benefits of peer feedback in the academic writing context were delineated and subsequently categorized into five categories: affective benefits, cognitive benefits, behavioral benefits, social benefits, and meta-cognitive benefits. Furthermore, an analysis of the implementation challenges revealed 13 types of obstacles, which were traced to three primary sources: challenges originating from feedback receivers, those posed by feedback providers, and those stemming from the peer feedback settings. Based on these findings, several pedagogical and future research suggestions are proffered to guide both the practitioners and researchers.

## 1 Introduction

In higher education, academic writing is considered a core competency for students (Chakraborty et al., 2021). Effective academic writing as the currency of intellectual exchange, which facilitates the sharing of novel insights and contributes to the advancement of knowledge, is crucial for the students’ academic success and career development (Aitchison and Lee, 2006; Swales and Feak, 1994). To date, significant emphasis has been placed on the academic writing instruction (Schillings et al., 2023). Defined as a process whereby students critically assess the level, merit, or quality of their peers’ work (Topping, 2009), peer feedback has garnered significant attention in recent years as an active learning strategy that fosters interaction, collaboration, and reciprocal learning (Liu and Carless, 2006). The integration of peer feedback into academic development is underpinned by theoretical frameworks that emphasize the social nature of learning and the role of collaborative interactions in the development of cognitive and metacognitive skills, such as the Collaborative Learning Theory in social psychology Bruffee (1984) and Vygotsky’s (1978) sociocultural theory.

In recent years, there has been a surge of interest in the role of peer feedback in academic writing education. Numerous studies have validated the effectiveness of these diverse peer feedback practices in advancing academic writing development. Prominently, peer feedback serves as a catalyst for elevating students’ academic writing quality and refining their academic writing skills (Kostopoulou and O’Dwyer, 2021; Rodas and Colombo, 2021). Beyond this, by engaging students in the evaluation process, peer feedback fosters a deeper understanding of academic writing criteria, promotes self-reflection, and enhances critical and analytical skills (Boillos, 2024; Davis, 2014; Kostopoulou and O’Dwyer, 2021; Osman et al., 2022), empowering students to become more discerning consumers and producers of academic texts (Ciampa and Wolfe, 2023; Pugh and Veitch, 2019; Yu, 2019). Furthermore, the collaborative nature of peer feedback encourages a sense of academic community and belonging within the learning environment, which can positively impact students’ motivation and engagement in the writing process (Geithner and Pollastro, 2016; Yallop et al., 2021).

However, the implementation of peer feedback in the academic writing context is not without its challenges. Insufficient feedback proficiency and domain-specific knowledge often translate into unproductive and unreliable feedback (Ciampa and Wolfe, 2023; Colombo and Rodas, 2021; Kostopoulou and O’Dwyer, 2021; López-Pellisa et al., 2021; Xu and Zhang, 2023). Moreover, the potential for interpersonal friction arising from the delivery of critical feedback and the risk of providing inadequate feedback pose further obstacles (Cheong et al., 2023; Ciampa and Wolfe, 2023; Rodas and Colombo, 2021; Yu, 2021). Some students may experience anxiety and insecurity when engaged in peer feedback activities, as they highly value camaraderie and harmony within their reviewing group (Xu and Li, 2018; Xue et al., 2023). Additionally, given the complexity of academic writing, learners tend to harbor a lower level of trust in peer feedback, particularly when juxtaposed against instructor-led feedback, underscoring the need for strategic interventions to address these concerns (Eppler et al., 2021; Pugh and Veitch, 2019; Xu and Zhang, 2023).

Despite the significant contributions of prior research in elucidating the merits and obstacles associated with integrating peer feedback into academic writing, a notable limitation persists in that these studies have focused narrowly on isolated facets of these benefits and challenges. A comprehensive synthesis of the broader spectrum of benefits and challenges has not been realized. Given the complexities of identified challenges, a comprehensive understanding of the potential challenges associated with the implementation of peer feedback in academic writing is conducive to effectively leveraging its advantages in practical applications. Furthermore, acknowledging the heterogeneous nature of benefits and challenges as identified in prior research, there is a compelling need for a systematic synthesis and taxonomy. Such an endeavor would significantly enrich our understanding and inform both instructional strategies and future research endeavors in this domain.

In response to this research gap, the present systematic literature review aims to provide a systematic synthesis of the empirical evidence on the benefits and challenges of incorporating peer feedback into academic writing instruction. Additionally, it aspires to discern trends in this realm, thereby offering guidance to both practitioners and researchers alike. To achieve this, this review will be guided by the following research questions:

1. What are the primary features of contemporary research investigating the effects of peer feedback in academic writing?
2. What are the multifaceted benefits of incorporating peer feedback into academic writing education, and how do they contribute to student learning and development?
3. What are the primary challenges encountered in implementing peer feedback in academic writing, and how do they affect the feedback process and its outcomes?

## 2 Methods

This study employed a systematic review methodology which entails a systematic collection and synthesis of pertinent articles guided by specific research questions (Aromataris and Pearson, 2014; Pearson, 2004; Siddaway et al., 2019). This approach allows researchers to produce more comprehensive and reliable conclusions by integrating diverse findings from previous studies, thereby providing insights for further research and practical applications. Though it was originally developed in medical sciences (Chalmers et al., 2002), numerous studies in the field of education have also attested to its effectiveness and utility (Andrews and Harlen, 2006; Bearman et al., 2012; Davies, 2000; Martin et al., 2020).

### 2.1 Data collection

**Figure 1:**
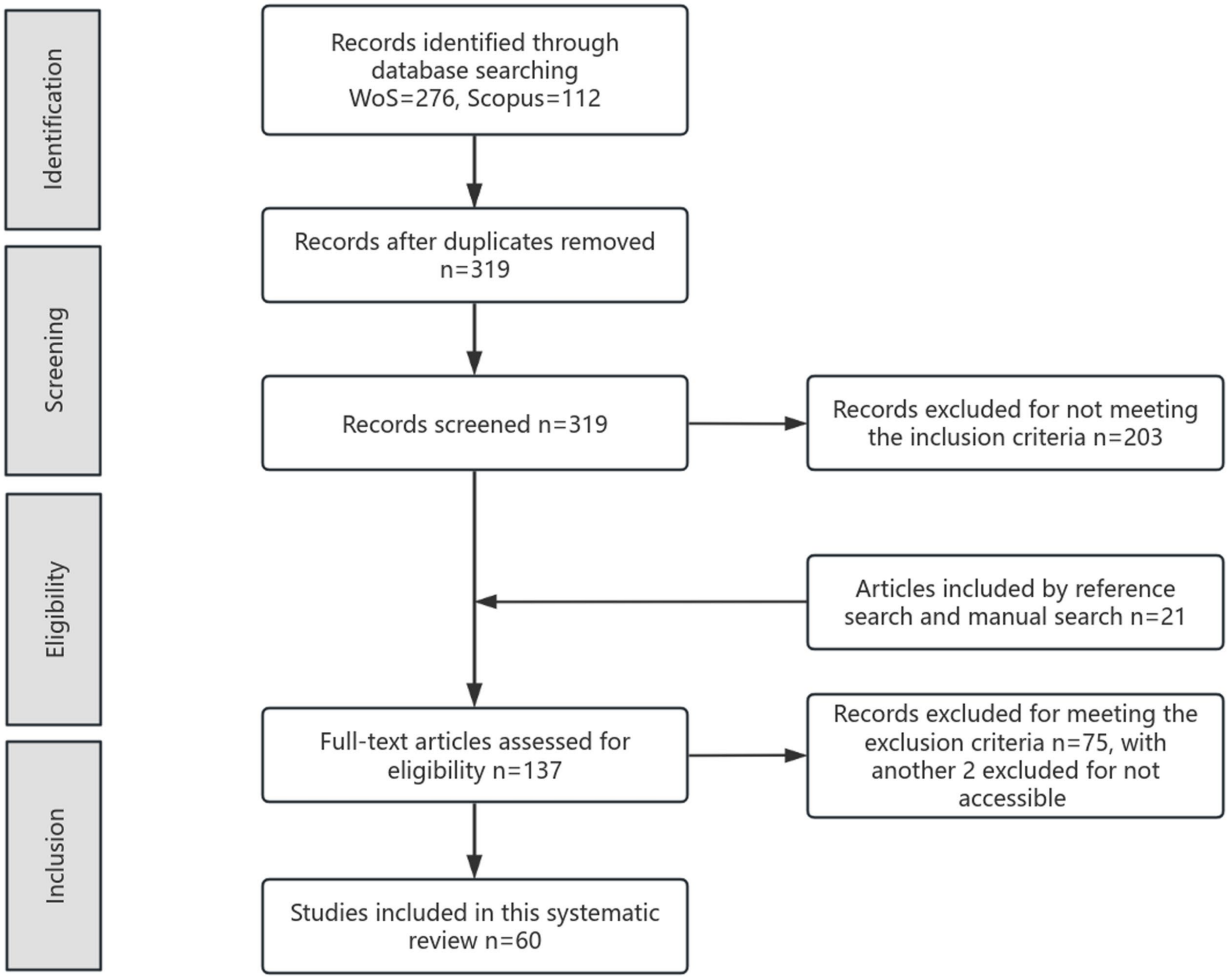
PRISMA flow diagram of the present review.

To guarantee the credibility of findings, this systematic review followed the guidance of PRISMA 2020 statement (Page et al., 2021), incorporating four stages of data collection: identification, screening, eligibility and inclusion. Details of this procedure are displayed in Figure 1.

In the identification of pertinent studies, three search methods were implemented: a database search, a reference search and a manual search. For the database search, Web of Science Core Collection and Scopus selected as sources owing to their esteemed reputation for encompassing extensive and high-caliber educational research. As a supplementary approach, the reference search was conducted to augment the search process by examining the cited references within the selected studies, thereby mitigating the risk of overlooking significant research contributions. Furthermore, a manual search was conducted utilizing Google Scholar as a platform to identify additional scholarly articles pertaining to the same subject matter.

Prior to embarking on a search for the relevant papers, index terms for the two main concepts, “academic writing” and “peer feedback,” were determined by inspecting search terms in previous review studies (Huisman et al., 2019; Yu and Lee, 2016; Zheng et al., 2019), and terminologies used in seminal and recent academic literature. This process resulted in 11 terms for “peer feedback”: peer assessment, peer feedback, peer review, peer evaluation, peer rating, peer scoring, peer grading, peer editing, peer response, peer interaction and student feedback, and three terms for “academic writing”: academic writing, research writing and scientific writing. These English terms were used in the search of relevant studies in Web of Science Core Collection, Scopus, and Google Scholar.

Regarding the concept of academic writing, the current study adopts the definition provided by Hyland (2004) and Swales (1990), which posits that academic writing constitutes the formal communication of research and ideas within a specific discipline, adhering to established conventions to contribute to and engage with the field’s knowledge. Therefore, this study focuses on various writing genres that differ from school writing, such as course essays, project reports, research proposals, lab notes, journal articles, conference papers, theses, and dissertations, as part of academic writing, regardless of whether they are written in a first or second language. The first author conducted the database search in July 2024, during which only peer-reviewed empirical studies published after 2013 were included for further examination. The initial literature search identified 276 articles from Web of Science Core Collection and 112 from Scopus. After removing 69 duplicates, 319 articles were selected for title and abstract screening to examine whether they meet the inclusion criteria. The following inclusion criteria were used to ensure the relevance and quality of selected articles: (1) published between 2014 and 2024; (2) empirical research; (3) articles concerning peer feedback to academic writing in higher education; (4) articles written in English. After that, 21 relevant papers identified by reference search and manual search were added to the results, which formed a refined pool of 340 articles for eligibility test through full text analysis. It was conducted under the guidance of following exclusion criteria: (1) articles not concerning peer feedback in higher education; (2) articles not revealing the benefits or the problems of peer feedback; (3) articles not clearly demonstrating the context of academic writing; (4) articles not involving peer feedback on their peer’s academic writing. Ultimately, this rigorous selection process yielded a total of 60 peer-reviewed empirical studies which were deemed most pertinent for investigating the multifaceted benefits and problems of peer feedback within the academic writing context. This process was visualized in Figure 1.

### 2.2 Data analysis

The present study employed a conventional content analysis (Hsieh and Shannon, 2005) to delve into the primary features of studies examining the effects of peer feedback in an academic writing context, as well as to identify and analyze the specific benefits and challenges that have been discerned. Conventional content analysis is an inductive category development method during which researchers refrain from relying on pre-determined categories and instead engage in an immersive process of data exploration, from which categories are derived (Kondracki and Wellman, 2002). It is typically deemed suitable in scenarios where the existing theoretical framework or research literature pertaining to a particular phenomenon is limited (Hsieh and Shannon, 2005), aligning well with the circumstances of the current study.

The collected data were scrutinized with the aim of analyzing the overarching themes and discernible patterns of the findings in the selected literature (Opfer and Pedder, 2011). Specifically, Microsoft Excel was employed to record and analyze the codes. To identify the primary features of the studies into the effects of peer feedback in academic writing, subcategories of descriptive information of the reviewed articles were analyzed. This encompassed an examination of the temporal distribution of reviewed studies by year, the research methodologies employed, the educational level of the participants, the subject domain of the academic writing, the task types involved, as well as the geographical locations and educational contexts within which these studies were undertaken (see Table 1). Moreover, contents regarding benefits and challenges of peer feedback underwent a three-stage analytical process. In the first stage, articles were coded using the words in the original text. As the author progressed through the data analysis, efforts were made to minimize the introduction of new codes, giving precedence to existing codes unless novel data emerged that could not be accommodated by them. Following the completion of coding all articles, a meticulous review of the data within each specific code was undertaken to explore potential combinations and segregations, which leads to the formation of distinct subcategories pertaining to benefits and challenges. To ensure reliability, the first author conducted two rounds of coding on all articles, with a two-month interval between the two coding sessions. The consistency rate of coding across all subcategories was not less than 93.3%, indicating a high level of reliability.

**Table 1:** Charting categories, subcategories, and description.

Categories	Subcategories	Description
Descriptive information	Year of publication	It refers to the year when the article was officially published in print, except in cases where it is exclusively published digitally.
	Country of article	It refers to country where the study was conducted.
	Research methodology	It refers to the methodology used in the article, including qualitative, quantitative, and mixed methodology.
	Sample group	It refers to the educational level of the participants, such as undergraduate students, master’s students, and doctoral students.
	Subject domain	It refers to the subject domain of the academic writing, such as natural science, social science, and engineering and technological science.
	Task types	It refers to the specific genre of academic writing, such as the scientific paper, scientific report, and research proposal.
Findings	Benefits	It refers to the benefits of incorporating peer feedback in academic writing.
	Challenges	It refers to the challenges encountered in incorporating peer feedback in academic writing.

## 3 Findings

### 3.1 Primary features of the reviewed articles

**Figure 2:**
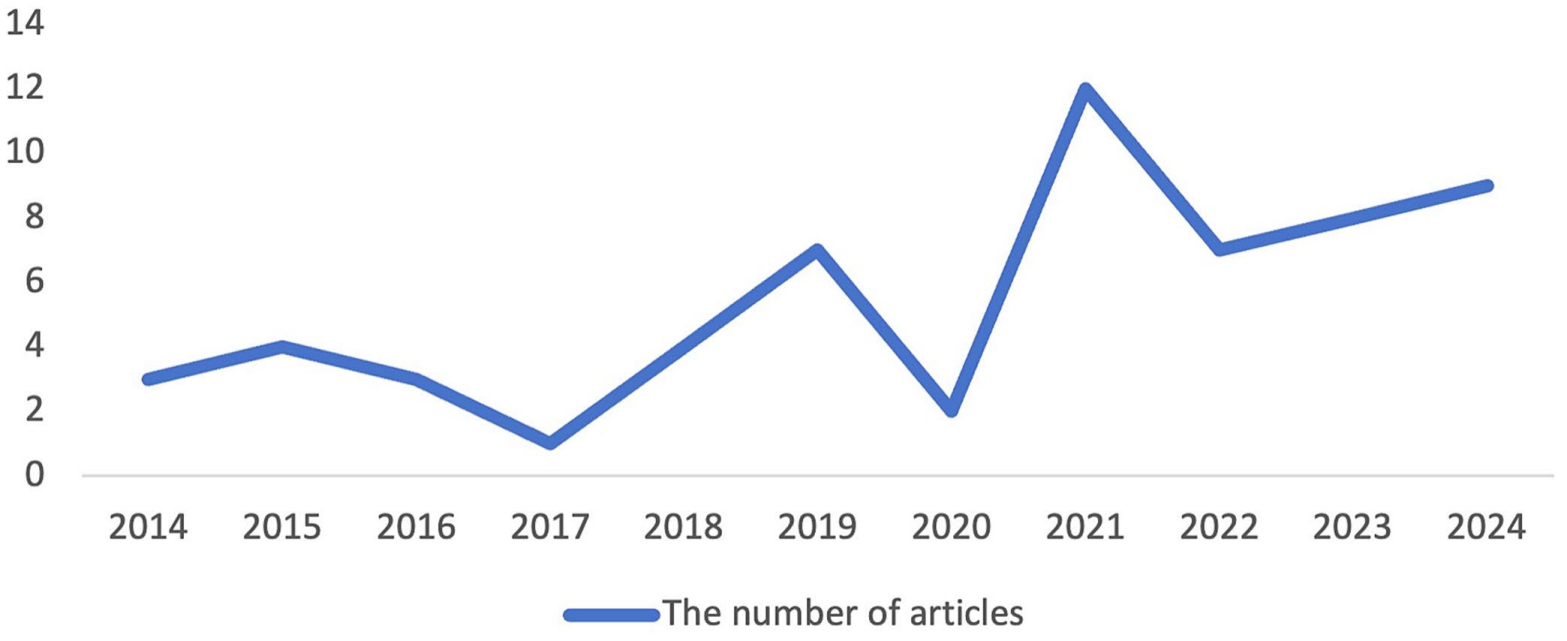
Temporal distribution of reviewed articles by year.

Figure 2 depicts the annual temporal distribution of the reviewed studies, illustrating the evolution of research endeavors over time. Prior to 2014, scholarly investigation into the effects of peer feedback within the context of academic writing had already emerged. Despite fluctuations observed in the escalating engagement within this domain, the past 3 years have exhibited a heightened level of interest compared to previous years. Given that the data was collected in July 2024, it is anticipated that the count of related articles for the year 2024 will surpass nine, signifying a substantial growth trend.

The methodologies employed across the reviewed literature were scrutinized. It was found that the mixed methodology emerges as the most prevalent approach (*n* = 24), closely followed by the qualitative methodology (*n* = 22). Conversely, the quantitative methodology is the least utilized (*n* = 14).

Moreover, the reviewed articles have investigated the effects of peer feedback in academic writing context utilizing data sourced from participants with various educational levels, such as undergraduates, master’s students and doctoral students. Predominantly, these studies have focused on examining the effects of peer feedback on undergraduates’ academic writing (*n* = 30), comprising approximately 50% of the reviewed corpus. Comparable emphasis has been placed on master’s students (*n* = 18) and doctoral students (*n* = 19), with a marginal increase in attention toward the latter. Moreover, a subset of studies (*n* = 6) has extended its scope to include participants from alternative educational levels, for instance, pre-master and pre-bachelor programs. It is pertinent to clarify that when studies encompass participants spanning multiple educational levels, they are accounted for within each respective subgroup, thereby leading to a cumulative total of subgroups exceeding the overall count of reviewed articles. The same calculating method is employed in the examination of subject domain, task type and country.

**Figure 3:**
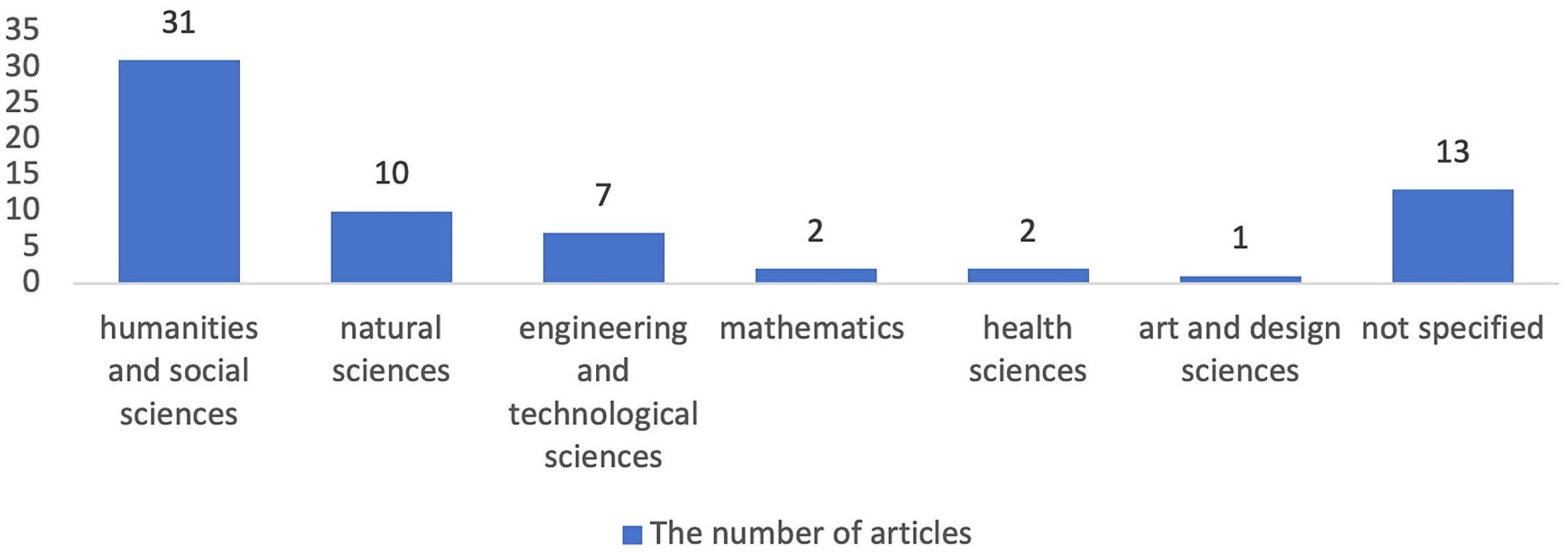
Subject domain of the academic writing.

**Figure 4:**
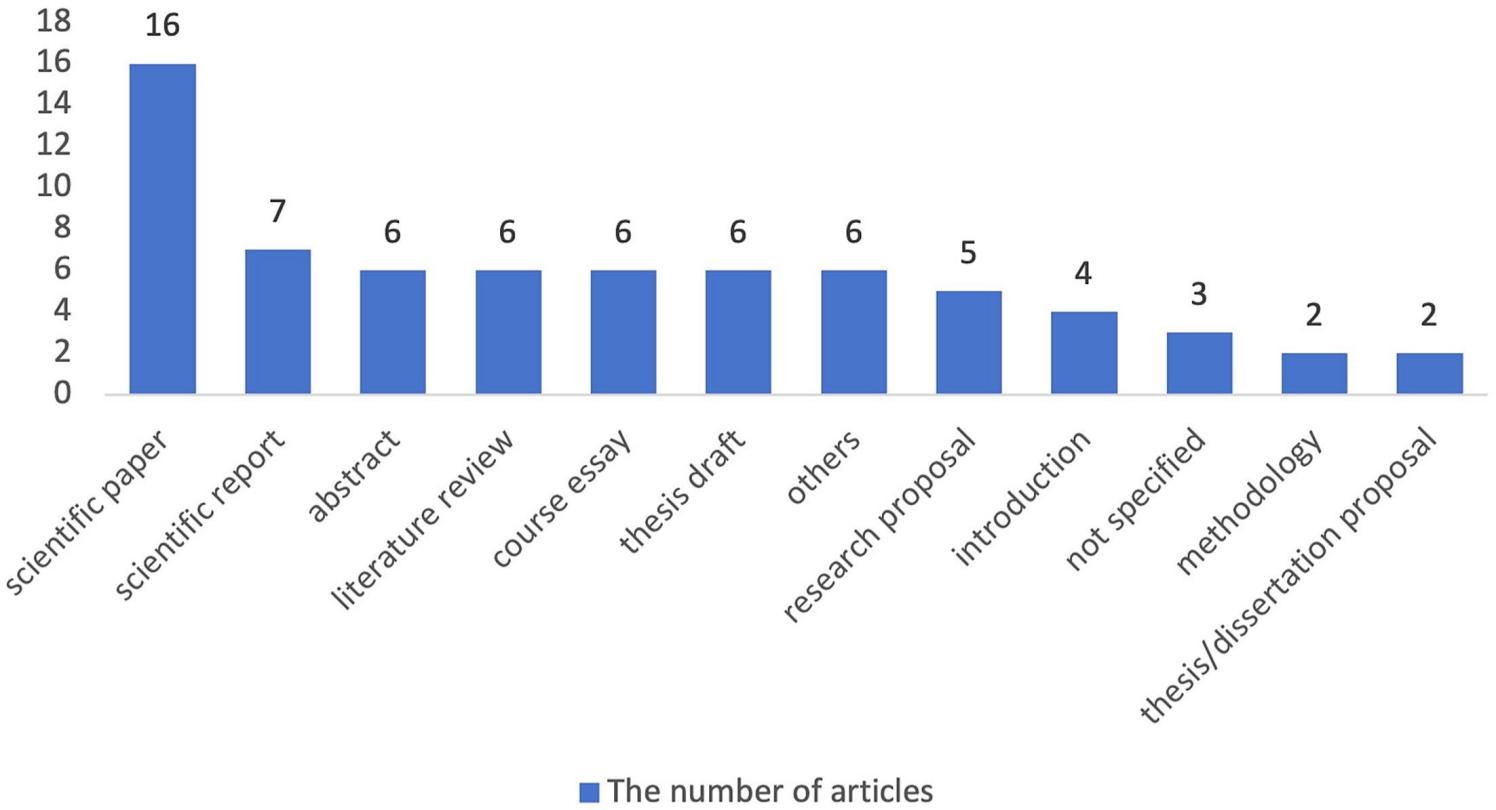
Task types of academic writing.

The subject domains and task types of the academic writing in the reviewed articles are visually depicted in Figures 3, 4 respectively. The utilization of peer feedback as a strategy in the development of academic writing has been observed across a diverse spectrum of subject domains. Notably, this approach was the most prevalent in the humanities and social sciences (*n* = 31), significantly outnumbering its application in natural sciences (*n* = 10) and engineering and technological sciences (*n* = 7), which occupy the second and third positions, respectively. Marginal but noteworthy attention was also accorded to the academic writing context within mathematics (*n* = 2), health sciences (*n* = 2), and art and design sciences (*n* = 1). Furthermore, an additional 13 articles existed that did not explicitly delineate the subject domain within which academic writing was being addressed. Regarding the distribution of task types, scientific papers constitute the most frequently encountered academic writing assignment within the reviewed articles (*n* = 16), followed by scientific reports, which represent a much smaller proportion (*n* = 7). Beyond these general categories, a notable number of studies delved into the writing of specific components within a scientific paper, specifically abstracts (*n* = 6), introductions (*n* = 4), methodologies (*n* = 2), and literature reviews (*n* = 6). Furthermore, the scope of academic writing examined also encompassed thesis/dissertation-related works, more specifically thesis/dissertation proposals (*n* = 2) and thesis drafts (*n* = 6). Additionally, research proposals (*n* = 5) and course essays (*n* = 6) also received similar attention.

**Figure 5:**
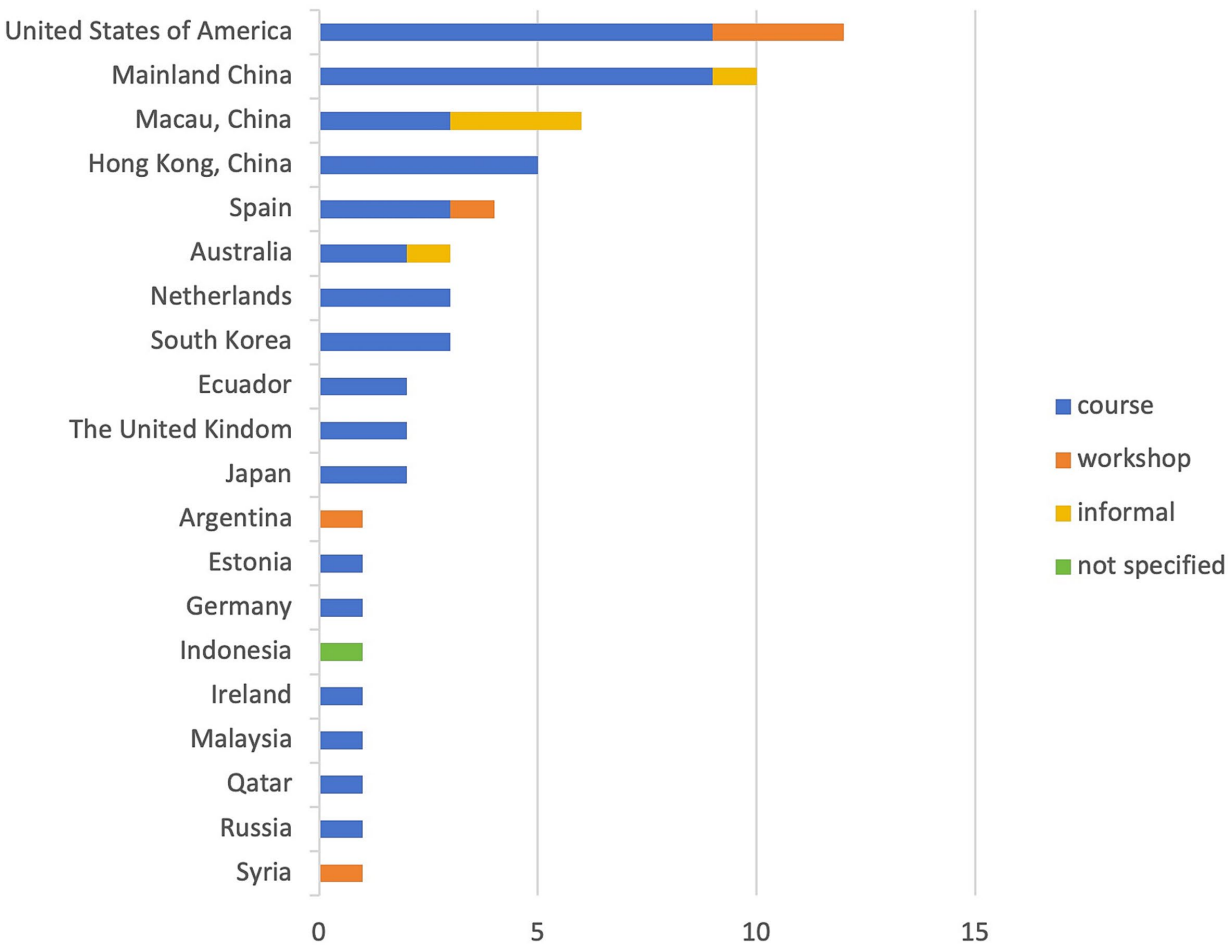
Countries (regions) and educational contexts of the reviewed articles.

Figure 5 delineates the countries (regions) and educational contexts within which these investigations into the integration of peer feedback in academic writing development were conducted. This strategy was observed to be embraced across a diverse range of countries and regions (*n* = 20), underscoring its widespread popularity in the realm of academic writing instruction. Notably, the preponderance of related research was situated in the United States (*n* = 12) and within China, encompassing mainland China (*n* = 10), Macau (*n* = 6), and Hong Kong (*n* = 5), collectively accounting for 55% of the total reviewed articles. In terms of educational contexts, three distinct modalities were identified for the implementation of peer feedback: courses, workshops, and other informal settings, such as self-organized writing groups. A dominant proportion of the studies were carried out within the structured environment of formal courses (*n* = 48, 81.7% of the total), with a minority being conducted in workshop settings (*n* = 6) and within informal contexts (*n* = 5). Notably, the examination of peer feedback’s effects in workshop settings was confined to a limited number of countries, including the United States (*n* = 3), Spain (*n* = 1), Argentina (*n* = 1), and Syria (*n* = 1). Conversely, no studies examining peer feedback in workshop contexts were found to have been conducted in China.

### 3.2 Specific benefits and challenges identified in previous studies

After conducting a content analysis of the reviewed literature, this study uncovered both the benefits and challenges associated with integrating peer feedback into academic writing. These findings are systematically organized and presented in Tables 2, 3.

**Table 2:** Benefits of incorporating peer feedback in academic writing.

Categories of benefits	Subcategories of benefits	Number of articles	Sample article
Cognitive benefits	Improving critical and analytical skills	15	Osman et al. (2022)
	Improving academic writing skills	11	Ramon-Casas et al. (2019)
	Knowing more about peer review process	6	Eppler et al. (2021)
	Developing communication skills	5	Gumusoglu et al. (2022)
	Developing feedback literacy	3	Wu and Lei (2023)
	Strengthening subject knowledge	1	Goh et al. (2019)
Behavioral benefits	Improving writing quality	33	Shulgina et al. (2024a)
Affective benefits	Strengthening confidence in academic writing	9	Xu and Zhang (2023)
	Strengthening confidence in critically analyzing academic work	5	Davis (2014)
	Improving willingness to ask for help in the future	1	Liu et al. (2021)
	Increasing motivation toward academic writing	1	Yallop et al. (2021)
Social benefits	Constructing academic community	7	Man et al. (2018)
	Gaining social support	4	Santelmann et al. (2018)
	Strengthening interpersonal relationship	2	Liu et al. (2021)
Meta-cognitive benefits	Promoting self-reflection	16	Yu (2019)
	Increasing metacognitive awareness of the writing process	9	Santelmann et al. (2018)

**Table 3:** Challenges in incorporating peer feedback in academic writing.

Categories of challenges	Subcategories of challenges	Number of articles	Sample article
Challenges from peer feedback receivers	Inadequate feedback literacy	7	Wu and Lei (2023)
	Negative attitude to peer feedback	5	Álvarez et al. (2015)
	Individual difference in gaining benefits	4	Ramon-Casas et al. (2019)
	Heavy cognitive load	2	Shulgina et al. (2024b)
	Low text quality	1	Pugh and Veitch (2019)
	Dependence on peer feedback	1	Lu et al. (2023)
Challenges from peer feedback providers	Students’ deficiency in providing constructive feedback	21	Cheong et al. (2023)
	Lack of confidence in providing constructive feedback	6	Ciampa and Wolfe (2023)
	Disregarding providing feedback	5	Xu and Li (2018)
Challenges from peer feedback settings	Interpersonal concerns	8	Zhang et al. (2022)
	Problems of distracting factors	2	Ahmed and Al-Kadi̇ (2021)
	Ineffective grouping of peers	2	Ahmed (2021)
	High time demand	1	Ahmed (2021)

The benefits, in particular, were classified into five distinct categories, each corresponding to a specific facet of enhancement: cognitive, behavioral, affective, social, and meta-cognitive benefits. Cognitive benefits pertain to the development of intellectual abilities such as thinking, knowledge representation, information processing, and decision-making, which are essential for the construction of knowledge during the learning process (Liu et al., 2022; Potvin et al., 2018; Svalberg, 2009; Swain, 2013). Behavioral benefits are associated with the positive changes in students’ external actions and academic activities (Nazamud-din et al., 2020; Uher, 2016). Affective benefits relate to the positive influence of peer feedback on students’ emotional experiences, including their confidence, willingness, and motivation (Gondim and Mutti, 2011; Nazamud-din et al., 2020; Piaget, 1962). Social benefits are linked to the positive effects of peer feedback on student interactions within the context of language learning (Svalberg, 2009). Lastly, metacognitive benefits involve the enhancement of self-reflection and the ability to regulate cognition, which are critical for optimizing learning (Goupil and Kouider, 2019; Moses and Baird, 1999). In the reviewed articles, self-reflection and metacognitive awareness of the writing process, such as audience awareness and writer awareness, was found to be improved by the peer feedback practice.

The challenges of incorporating peer feedback in academic writing are found to have three sources: challenges from peer feedback receivers, challenges from peer feedback providers and challenges from peer feedback settings. Subcategories of them are presented in Table 3.

## 4 Discussion

### 4.1 Primary features of the research examining the effects of peer feedback in academic writing

The visualization of the temporal distribution of the reviewed articles exhibits a nuanced dynamic, wherein despite fluctuations in the annual count of related studies, a general trend of escalating interest in the subject matter is discernible over the years. Notably, the past 3 years have witnessed a sustained increase in the level of engagement with this topic. This can be attributed to the heightened emphasis accorded to academic writing within higher education (Chakraborty et al., 2021), and the burgeoning popularity of peer feedback mechanisms in academic writing development, along with their acknowledged merits (Boillos, 2024; Kostopoulou and O’Dwyer, 2021; Osman et al., 2022).

In terms of the research methodologies adopted within the corpus of reviewed articles, a comparative analysis reveals that mixed-methods and qualitative approaches occupy comparable and substantial proportions (40 and 37%, respectively), whereas quantitative methods are less prevalent, accounting for merely 23% (14 studies). Furthermore, despite over half of the reviewed studies incorporating quantitative data analysis, a closer inspection reveals that the majority of these studies focused exclusively on assessing the influence on enhancing writing quality, leading to a notable absence of quantitative data pertaining to other facets. This disparity underscores the need for a more robust quantitative interrogation to validate the discerned benefits and obstacles, thereby fostering a deeper understanding of the topic.

Regarding the educational levels of participants, half of the studies examined undergraduates, whereas doctoral and master’s students were involved in 32 and 30% of the research samples, respectively. This preponderance of undergraduate focus likely stems from the heightened importance attributed to academic writing instruction at the undergraduate level within higher education systems. Conventionally, it is assumed that postgraduates, having completed their undergraduate studies, possess a foundational proficiency in academic writing (Sallee et al., 2011; Singleton-Jackson and Lumsden, 2009). However, research findings challenge this notion, revealing that post-graduates often encounter challenges in academic writing and continue to require instructional support (Santelmann et al., 2018; Kabaran, 2022). In light of this revelation, further investigations are imperative to delve into the effectiveness of peer feedback mechanisms for postgraduate students. Such studies would not only elucidate the specific impact of peer feedback on enhancing postgraduate academic writing but also facilitate the strategic integration of this method into the development of postgraduate writing competencies, ultimately contributing to the holistic advancement of academic writing skills across all levels of higher education.

In terms of the subject domains of academic writing, a discernible hierarchy emerges, with humanities and social sciences (*n* = 31) occupying the foremost position, followed by natural sciences (*n* = 10), and engineering and technological sciences (*n* = 7). Conversely, mathematics (*n* = 2), health sciences (*n* = 2), and art and design sciences (*n* = 1) received comparatively limited attention. This distribution may be attributed to the substantial student enrollment in the aforementioned major disciplines, along with their relatively greater accessibility. Furthermore, an analysis of the task types in the reviewed articles reveals a predilection toward scientific papers and their constituent elements. Specifically, the abstract, introduction, literature review, and methodology garnered exceptional emphasis, likely stemming from their pivotal role in shaping the integrity and rigor of a scientific paper. This underscores the criticality of these components in contributing to the overall quality and comprehension of scientific research.

The analysis of national landscapes and educational contexts within the reviewed articles underscores the widespread adoption of peer feedback as a strategy for enhancing academic writing capabilities across diverse countries, spanning from the United States of America to Syria. This trend underscores the popularity and efficacy of peer feedback in fostering academic writing development (Kostopoulou and O’Dwyer, 2021; Rodas and Colombo, 2021). The United States leads the way in research endeavors, with the highest number of studies conducted (*n* = 12), closely followed by mainland China (*n* = 10), Macau China (*n* = 6), and Hong Kong China (*n* = 5). This distribution indicates that both the United States and China prioritize peer feedback as a vital tool in nurturing academic writing skills. However, it is noteworthy that the majority of the reviewed studies (82%) implemented peer feedback within the confines of formal coursework. Despite China’s significant contribution to the field, notably absent are studies examining peer feedback in a workshop setting. This observation may be attributed to the fact that academic writing instruction is predominantly conducted within classroom environments, whereas workshop organizers may not fully recognize the inherent value of peer feedback as an instructional method.

### 4.2 Benefits and challenges identified in incorporation peer feedback in academic writing

In the examination of the reported benefits of incorporating peer feedback in academic writing, various benefits have been identified which can be divided into five categories, namely, cognitive benefits, behavioral benefits, affective benefits, social benefits, and meta-cognitive benefits. Among these, at the macro-level, cognitive benefits are the most frequently reported, followed closely by behavioral and meta-cognitive benefits.

Among the specific benefits identified, the most frequently reported one is the behavioral benefit of stimulating revisions to their academic work, ultimately yielding a positive outcome in the form of enhanced writing quality. For instance, Lineback and Holbrook (2023) conducted a rigorous investigation utilizing both qualitative and quantitative methodologies to assess the differences between pre-draft and post-draft versions of students’ work. Their analysis encompassed a statistical examination of the scores and an in-depth exploration of students’ revision processes and the peer feedback received. The results of this study revealed that 14 out of 15 students experienced an improvement in their overall scores, with 13 students implementing at least one discernible change that could be directly attributed to the influence of peer feedback. Furthermore, in the investigation of the precise domains exhibiting enhancement subsequent to peer feedback, research has demonstrated that the enhancement of academic writing quality through revision extends to multiple dimensions of academic writing, including but not limited to, the refinement of organizational structure (Kostopoulou and O’Dwyer, 2021; Rodas and Colombo, 2021), the accuracy and appropriateness of in-text citations (Kostopoulou and O’Dwyer, 2021), the depth and clarity of ideas and content (Boillos, 2024; Greenberg, 2015), as well as linguistic precision and appropriateness (Kostopoulou and O’Dwyer, 2021; Zhang et al., 2020).

The second most prevalent advantage, as reported, lies in the meta-cognitive enhancement of self-reflection. Participants generally reported that engaging in peer feedback elicited self-reflection and fostered a more reflective learning approach (Pugh and Veitch, 2019). More precisely, the activity of comparing papers written by different individuals and discussing issues during the peer feedback process prompted students to reflect on their academic writing, with the former activity often functioning spontaneously (Deng et al., 2019). For instance, through the utilization of data sourced from interviews and stimulated recall techniques, Yu’s research examining the experiences associated with peer feedback practices during the process of master’s thesis writing elucidates that engagement in peer feedback fosters self-reflection upon one’s own writing. Through reflection subsequent to critical peer feedback, students strengthened their critical thinking ability and developed into critical readers and writers of academic literature (Ciampa and Wolfe, 2023; Yu, 2019).

Furthermore, some studies have documented that peer feedback activity significantly contribute to the enhancement of students’ critical and analytical skills (e.g., Osman et al., 2022), and the confidence in providing constructive peer feedback (Davis, 2014). More specifically, peer feedback practice equips learners with the capacity and confidence to engage in a critical assessment of both their own and their peers’ academic work (Boillos, 2024; Davis, 2014; Geithner and Pollastro, 2016; Schillings et al., 2021). For instance, participants in Geithner and Pollastro’s (2016) study rated their “ability to provide peer review” significantly higher subsequent to peer feedback practice. Notably, four articles have underscored the superiority of public multi-peer feedback in fostering these essential skills. Specifically, these studies reveal that the diverse perspectives accessible to individual students within the framework of public multi-peer feedback facilitate the identification of overlooked aspects in their own feedback practices, thereby facilitating the refinement and honing of their analytical skills (Gao and Chen, 2024; Chen and Gao, 2024). This underscores the importance of such collaborative feedback mechanisms in nurturing critical thinking and analytical proficiency among students.

It is also noteworthy to highlight the convergence of seven articles, which affirm that the integration of peer feedback into the academic writing process constitutes a significant contributor to the construction of an academic community. Specifically, the interactive exchange during peer feedback sessions, particularly the affective devices embedded in comments, fosters a sense of community among students (Yallop et al., 2021). Furthermore, this practice facilitates the introduction of graduate students into established scholarly networks (Ciampa and Wolfe, 2023; Man et al., 2018; Zhang et al., 2020). For example, Man et al. (2018) examined autonomous peer feedback practices among postgraduate students and observed that such feedback not only catalyzes the construction of new academic communities but also facilitates the introduction of graduate students into established scholarly networks, echoing the findings of Ciampa and Wolfe (2023) as well as Zhang et al. (2020). In the academic community, peer feedback assumes a pivotal role, serving as a conduit for transmitting academic writing norms and nurturing interpersonal relationships (Zhang et al., 2020). Notably, two recent studies have underscored the distinct advantages of community-based peer feedback in constructing academic community. They emphasized the capacity of this approach to forge social and emotional bonds among classmates, thereby fostering the formation of a cohesive academic community (Gao and Chen, 2024; Chen and Gao, 2024). This underscores the importance of peer feedback not merely as a technical tool but also as a catalyst for building a supportive and collaborative scholarly environment.

Despite many studies elucidating the favorable influence of peer feedback on the revision process, ultimately fostering the advancement of the current writing quality (e.g., Kostopoulou and O’Dwyer, 2021; Rodas and Colombo, 2021), a comparatively scarce body of research has explicitly documented the improvement in writing skills, as evidenced by students’ demonstrated capacity to produce high-quality academic writing. Furthermore, within the subset of studies offering such evidence, a substantial proportion relies on self-assessment as the primary metric. For example, López-Pellisa et al. (2021) utilized a 5-point Likert scale to investigate students’ perceived enhancement of academic writing proficiency through peer feedback activity. The results of their study indicate that a majority of the participants reported an improvement in their academic writing proficiency subsequent to peer feedback activity. A mere two studies examined the academic writing competence before and after the application of peer feedback (e.g., Hanafi et al., 2024; Ramon-Casas et al., 2019), thereby offering a more objective assessment of the skill enhancement. This paucity of research underscores the need for further investigation to evaluate the impact of peer feedback on the development of academic writing skills.

Despite the multifaceted benefits associated with integrating peer feedback into academic writing, this practice also encounters some challenges, which can be systematically categorized into three distinct categories based on their origins: challenges from peer feedback receivers, challenges from peer feedback providers, and challenges from peer feedback settings.

At a macroscopic level, research has predominantly documented challenges emanating from feedback providers, with subsequent emphasis on those confronted by peer feedback recipients, and finally, challenges inherent in the peer feedback settings. This hierarchical pattern underscores the pivotal role of the two fundamental components of peer feedback activities—the providers and receivers—as the primary sources of issues encountered within this educational practice.

Among the subcategories of challenges, students’ deficiency in providing constructive feedback emerges as the paramount obstacle. This underscores a pervasive inability among students to provide insightful peer feedback, a challenge that has been consistently noted across diverse educational contexts, encompassing undergraduate and graduate students at various stages of their academic journey (Álvarez et al., 2015; Gumusoglu et al., 2022). Specifically, feedback was noted to be either insufficient (López-Pellisa et al., 2021; Xu and Zhang, 2023), or characterized by over-generalization, brevity, and superficiality (Cheong et al., 2023; Weaver et al., 2014), neglecting the intricate issues that truly require attention (Gao et al., 2019). Notably, the quantitative data in Cheong et al.’s (2023) study revealed that 60.8% of the suggestions in peer feedback lacked specificity, minimally contributing to the revision process. This issue can be attributed, in part, to the intricate cognitive and social processing skills required for effective peer feedback generation (Xu and Zhang, 2023), rendering it a formidable task for students to generate constructive feedback on their peers’ manuscripts. Furthermore, factors such as limited experience (Yucel et al., 2014), inadequate subject knowledge (Kostopoulou and O’Dwyer, 2021), and constrained metacognitive abilities (Nur and Anas, 2022) have also been identified as contributing factors to this challenge. This deficiency has the potential to engender a lack of trust among students in receiving constructive feedback from their peers (Jurkowski, 2018; Pugh and Veitch, 2019), ultimately impairing their engagement and reducing the efficacy of the peer feedback practice (Álvarez et al., 2015; Jurkowski, 2018).

Consistent with the observed deficiency in delivering constructive feedback, six articles have documented a prevalent lack of confidence among students in providing peer feedback. Notably, Yu’s (2019, 2021) research revealed that, despite their enthusiasm for engaging in peer feedback tasks, master’s students harbored doubts regarding their linguistic competence, the accuracy and constructiveness of their feedback, and the interpersonal skills necessary for effective peer feedback. Similarly, Ciampa and Wolfe’s (2023) study found that doctoral students, despite being advanced academic writers, struggled with perceived inadequacy in their expertise and experience, leading to similar confidence issues. These findings underscore the widespread occurrence of confidence deficits across different academic levels in the context of peer feedback on academic writing. Addressing these confidence deficits is crucial, as they can significantly hinder students’ participation in peer feedback, thereby undermining the overall effectiveness of this pedagogical practice (Allen and Katayama, 2016; Xu and Li, 2018; Xue et al., 2023).

Eight articles have consistently highlighted the second most prevalent challenge, which revolves around students’ interpersonal apprehensions in offering constructive critiques on their peers’ academic writings in non-anonymous settings. Notably, a preponderance of these investigations (specifically, five out of the eight studies) was situated within the Chinese cultural context, where an emphasis on maintaining a harmonious environment is deeply ingrained (Xu and Li, 2018). This psychological pressure can subsequently precipitate a reluctance among students to engage in the peer feedback process (Xu and Li, 2018), or prompt them to grant overly generous grades to their peers (Cheong et al., 2023). For instance, Zhang et al. (2022) reveal that the “face”-threatening dilemmas in the Chinese context cultivate a tendency among students to preserve interpersonal harmony, which often entails an aversion to losing face for their peers. Consequently, it undermines trust among peers and negatively impacts students’ willingness to provide constructive feedback, thereby hindering the overall effectiveness of this pedagogical approach.

The deficiency in feedback literacy among receivers also emerges as a significant obstacle in the integration of peer feedback within the domain of academic writing. Feedback literacy, as defined by researchers such as Carless and Boud (2018), encompasses a deep understanding of feedback and effective management, the capacity and disposition to leverage feedback, as well as an appreciation of the roles of teachers and students themselves in this process. Studies have revealed a tendency among students to selectively incorporate feedback, giving priority to simpler suggestions over more complex ones when revising their academic writing (Shulgina et al., 2024a; Gao et al., 2019; Zhang et al., 2020). For example, in the study of master’s students’ revision processes, Zhang et al. found a lower revision rate for content-focused feedback (86.11%) compared to form-focused feedback (97.56%). This phenomenon echoes Yu et al.’s (2019) findings, where despite significant behavioral engagement, students lacked strategies and meta-cognitive processing of the feedback, resulting in superficial engagement that hindered the productive use of feedback. These insights emphasize the need for targeted interventions aimed at enhancing students’ feedback literacy. By improving their understanding of feedback, empowering them to leverage it effectively, and fostering an appreciation for the peer feedback process, educators can help ensure that the potential of peer feedback is realized in fostering the development of academic writing skills among students.

### 4.3 Pedagogical implications

The literature under review underscores the multifarious advantages of integrating peer feedback into the process of academic writing. These benefits encompass a broad spectrum, including cognitive enhancements that facilitate academic writing and critical analysis; behavioral improvements marked by active revision in the writing task; affective gains in the form of enhanced self-confidence, heightened willingness, and increased motivation; social benefits stemming from collaborative learning, and a sense of community among peers; as well as meta-cognitive benefits, which are characterized by intensified self-reflection and a heightened meta-cognitive awareness of the writing process, enabling students to better understand and regulate their own writing strategies and approaches. This comprehensive array of benefits underscores the justification for incorporating this method in the development of academic writing.

However, this approach also encounters many challenges, with the most salient being students’ deficiency in providing constructive peer feedback, inadequate feedback literacy, and the interpersonal concerns in offering constructive critiques on their peers’ academic writings. Drawing upon the insights garnered from the reviewed literature, the subsequent pedagogical interventions are proposed as potential solutions.

Firstly, the provision of comprehensive training on giving peer feedback is paramount to enhancing the overall effectiveness of this practice (Lu et al., 2021; Pugh and Veitch, 2019). Academic writing is an advanced type of writing distinct from conventional school writing, which necessitates a profound grasp of disciplinary knowledge and genre-specific competencies. Students often grapple not just with superficial aspects like vocabulary and grammar but also with advanced facets of academic writing (Gao et al., 2019), including research methodology and the significance of research (Man et al., 2018). However, as evidenced in prior studies, peer feedback tends to focus predominantly on superficial issues, neglecting the more advanced aspects that are central to the purpose of peer feedback in academic writing instruction. Therefore, this training is vital to ensuring that students reap the full benefits of this practice (Chang, 2015; Liou and Peng, 2009).

Extending the discourse, an optimal peer feedback training program for reviewers ought to embody three fundamental elements: the clarification of reviewing criteria (Pugh and Veitch, 2019), the provision of exemplary feedback (Kostopoulou and O’Dwyer, 2021), and the cultivation of a conducive mindset (Yucel et al., 2014). To ensure that reviewers possess a foundational understanding of the pivotal aspects of academic writing, the development of rubrics from the outset is imperative. Rubrics serve as a catalyst for reviewers’ engagement in peer feedback (Yu, 2021), enhance their genre-specific knowledge and enable them to generate constructive critiques of their peers’ academic work (Ciampa and Wolfe, 2023; Tai et al., 2018). Prior research underscores the positive impact of rubrics in this regard (Ciampa and Wolfe, 2023; Greenberg, 2015; López-Pellisa et al., 2021; Yu, 2021).

A dedicated discussion session focusing on the specific items outlined in the rubrics is recommended. This forum fosters a deeper comprehension of the criteria among students (Yucel et al., 2014), encourages the sharing of insights, and promotes mutual learning (Yu et al., 2019). Additionally, the presentation of exemplary peer feedbacks is vital in illustrating the ideal form of constructive criticism (Costley et al., 2023; Shulgina et al., 2024b). This process should include detailed guidance on feedback-giving strategies, which encompassing emphasizing the importance of addressing advanced issues in academic writing (Gao et al., 2019), prioritizing quality over quantity (Shulgina et al., 2024b), offering comments rather than direct editing (Shulgina et al., 2024a), presenting a diverse range of feedback types that form a coherent logical structure (Lu et al., 2021), and attending to the manner in which feedback is delivered (Lu et al., 2023; Yallop et al., 2021; Zhang et al., 2020).

Furthermore, nurturing a favorable mindset among students is crucial for facilitating the peer feedback process in academic writing context (Yucel et al., 2014). Educators should underscore the potential benefits of both giving and receiving peer feedback, even when students are paired with less proficient peers (Shulgina et al., 2024a). This approach motivates students to engage positively in the activity and helps them establish realistic expectations (Yucel et al., 2014). By addressing these three elements comprehensively, an effective peer feedback training program can be established, thereby maximizing the benefits of this pedagogical practice.

Beyond the refinement of students’ feedback skills, an equally pivotal aspect is the cultivation of their feedback literacy, which ultimately determines their ability to reap the full benefits of peer feedback activities (Handley et al., 2011). Therefore, prior to engaging in peer feedback, students must be equipped with strategies to effectively leverage the feedback received (Lu et al., 2021; Yu et al., 2019). This involves teaching them how to incorporate suggestions into their revisions (Álvarez et al., 2015; Shulgina et al., 2024b), fostering feedback acceptance (Lu et al., 2023), and managing diverse types and volumes of feedback effectively (Lu et al., 2023; Shulgina et al., 2024b). Furthermore, students should be guided to participate in the peer feedback process with affective, behavioral, and cognitive engagement to maximize its benefits (Yu et al., 2019).

Although interpersonal concerns frequently emerge in non-anonymous contexts (e.g., Xue et al., 2023; Zhang et al., 2022), the decision to employ anonymity should also go through meticulous consideration. It was found that the anonymity of peer feedback can also deprive opportunities for face-to-face dialogue, which is vital for elaborating on feedback, fostering constructive commentary, and fostering a sense of responsibility (Schillings et al., 2021). Dialogue not only aids the peer feedback process in clarifying cognitive conflicts (Wu and Lei, 2023), but also in supporting students emotionally (Lineback and Holbrook, 2023), thereby facilitating the revision process. Therefore, alternative methods to mitigate interpersonal tension should be prioritized over anonymous feedback designs. For instance, teachers can impart communication skills that help students manage potentially negative emotions (Zhang et al., 2022).

### 4.4 Implications on future research

While prior research has provided valuable insights into the benefits and challenges of peer feedback within the realm of academic writing development, its scope is inherently limited in at least four key dimensions.

Firstly, considering the biased attention accorded to undergraduate students in this realm, it is imperative to embark on research endeavors directed toward master’s and doctoral students, with the aim of delving into the nuanced potential of peer feedback in academic writing instruction. These studies would not merely elucidate the intricate effects of peer feedback on augmenting postgraduate academic writing skills but also pave the way for a strategic integration of this approach into the development of writing competencies among postgraduate students. Ultimately, such endeavors would contribute significantly to informing the pedagogical implementation of peer feedback in academic writing practices across the entire spectrum of higher education.

Secondly, as highlighted in the preceding section, the quantitative evidence pertaining to the impact of peer feedback remains scarce. Consequently, future research endeavors ought to delve into this topic by conducting rigorous analyses of diverse quantitative datasets, with the aim of providing a more comprehensive and robust understanding of the effects of peer feedback on academic writing development.

Thirdly, while many studies have identified the positive effects of peer feedback on enhancing writing quality, a notable scarcity persists in long-term empirical evidence on the improved writing skills. However, the sustainability and transferability of these effects in fostering students’ writing abilities constitute a pivotal aspect in assessing the overall effectiveness of peer feedback (Zhang, 2021). Therefore, future research endeavors ought to employ lagged test to scrutinize the longitudinal effects, thereby elucidating the positive influence of peer feedback on students’ academic writing skills.

Lastly, the critical role of sociocultural factors in shaping students’ academic development is widely acknowledged, yet previous investigations into peer feedback in academic writing have been notably inadequate in this regard. Out of the reviewed articles, merely five have addressed the influence of cultural factors, and all are confined to the Chinese context, such as the concept of “face” (Zhang et al., 2022) and the tradition of harmonious communication (Xue et al., 2023). Studies that delve into diverse sociocultural backgrounds are expected to contribute significantly to our understanding of this issue, facilitating cross-cultural comparisons and the identification of both similarities and differences in the peer feedback process.

## 5 Conclusion

The current study employs the PRISMA framework for systematic review to scrutinize the utilization of peer feedback in academic writing. This approach not only maps out the prevailing trends in related research endeavors but also unveils benefits and challenges associated with this practice. The findings of this systematic review reveal a general upward trajectory in research interest, with investigations spanning multiple countries, attesting to the widespread adoption of peer feedback in academic writing pedagogy. However, a notable disparity exists, with a preponderance of studies centering on the formal classroom instruction of undergraduate students’ academic writing, as opposed to those focusing on master’s and doctoral candidates. Additionally, the preponderance of qualitative data employed in assessing the effects of peer feedback underscores the necessity for future research to adopt a quantitative lens, thereby enriching the understanding of this topic.

The integration of peer feedback in academic writing has been found to yield multifarious benefits, which can be categorized into five distinct domains: cognitive, behavioral, affective, social, and meta-cognitive. Notably, cognitive benefits emerge as the most frequently cited, followed by behavioral benefits, and meta-cognitive benefits. However, the implementation of this approach is not without its challenges, which can be traced to three primary sources: the receiver, the provider, and the setting. Key obstacles encountered include students’ inability to provide constructive feedback, a lack of feedback literacy, and interpersonal concerns associated with delivering critical comments. These challenges necessitate careful consideration and strategic interventions to ensure the effective utilization of peer feedback in academic writing instruction.

Despite the systematic review’s commendable effort in presenting the prevalent trend and synthesizing the effects of integrating peer feedback into academic writing instruction, thereby offering valuable guidance to both practitioners and researchers in the field, the current study notably confines its focus solely to synthesizing the outcomes of prior investigations. A more profound exploration of the interplay between the benefits and challenges is anticipated to yield more incisive insights.

## Data Availability

The original contributions presented in the study are included in the article/supplementary material, further inquiries can be directed to the corresponding author.
